# Morphological Characterization of *Cannabis sativa* L. Throughout Its Complete Life Cycle

**DOI:** 10.3390/plants12203646

**Published:** 2023-10-22

**Authors:** Mohsen Hesami, Marco Pepe, Andrew Maxwell Phineas Jones

**Affiliations:** Department of Plant Agriculture, University of Guelph, Guelph, ON N1G 2W1, Canada; mhesami@uoguelph.ca (M.H.);

**Keywords:** flowering, leaf morphology, phyllotaxy, phase transition, plant architecture, trichome, white widow

## Abstract

This study extensively characterizes the morphological characteristics, including the leaf morphology, plant structure, flower development, and trichome features throughout the entire life cycle of *Cannabis sativa* L. cv. White Widow. The developmental responses to photoperiodic variations were investigated from germination to mature plant senescence. The leaf morphology showed a progression of complexity, beginning with serrations in the 1st true leaves, until the emergence of nine leaflets in the 6th true leaves, followed by a distinct shift to eight, then seven leaflets with the 14th and 15th true leaves, respectively. Thereafter, the leaf complexity decreased, culminating in the emergence of a single leaflet from the 25th node. The leaf area peaked with the 12th leaves, which coincided with a change from opposite to alternate phyllotaxy. The stipule development at nodes 5 and 6 signified the vegetative phase, followed by bract and solitary flower development emerging in nodes 7–12, signifying the reproductive phase. The subsequent induction of short-day photoperiod triggered the formation of apical inflorescence. Mature flowers displayed abundant glandular trichomes on perigonal bracts, with stigma color changing from whitish-yellow to reddish-brown. A pronounced increase in trichome density was evident, particularly on the abaxial bract surface, following the onset of flowering. The trichomes exhibited simultaneous growth in stalk length and glandular head diameter and pronounced shifts in color. Hermaphroditism occurred well after the general harvest date. This comprehensive study documents the intricate photoperiod-driven morphological changes throughout the complete lifecycle of *Cannabis sativa* L. cv. White Widow. The developmental responses characterized provide valuable insights for industrial and research applications.

## 1. Introduction

*Cannabis sativa* L. (cannabis) is a multipurpose plant species known for its psychoactive and medicinal properties that has gained significant attention worldwide [1]. The increasing global acceptance of cannabis drives the necessity of scientific research aimed at understanding its biology for optimizing cultivation practices [2]. Cannabis contains a diverse array of chemical compounds, including cannabinoids, which are responsible for its therapeutic effects [3]. The concentration and composition of cannabinoids varies between cannabis cultivars, leading to distinct chemical profiles and differential biological effects [4]. In addition to cannabinoid content, morphological traits play a crucial roles in the overall physiology, yield potential, and quality of cannabis plants [5].

The morphological traits of cannabis encompass various aspects, including leaf morphology, plant architecture, and trichome characteristics [6]. Leaf morphology plays a crucial role in the overall physiology and performance of plants [7]. Cannabis leaves represent primary photosynthetic organs, where light is captured and converted into chemical energy [8]. The morphology of leaves, including their size, shape, and structure, directly influence the plant’s ability to efficiently capture light, exchange gases, and regulate transpiration [9]. Leaf morphology is tightly linked to key physiological processes such as water and nutrient uptake, carbon assimilation, and stress responses [10]. Moreover, leaf traits are often used as indicators of plant health, growth vigor, and productivity in agricultural and horticultural practices [7]. Understanding the emergent variations in cannabis leaf morphology and their implications can provide valuable insights for cultivation practices [6]. Therefore, comprehensive cannabis leaf morphology studies are essential in better understanding cannabis physiology for improved cultivation and medicinal applications.

The architecture of cannabis plants, including plant height, branching pattern, and overall structure, plays a significant role in determining their growth, productivity, and adaptability [6]. Cannabis exhibits considerable phenotypic plasticity, with variations in plant architecture observed among different cultivars and environmental conditions [6]. Plant height influences light interception, resource utilization, and biomass production, thereby impacting overall yield potential [11,12]. The branching pattern, including the number and arrangement of lateral branches, affects the distribution of resources, flower development, and cannabinoid synthesis [11]. The architecture of cannabis plants also influences pest and disease resistance, as well as the ease of cultivation, harvesting, and management practices [6]. Understanding the factors that influence cannabis architecture can inform producers to develop cultivars with desired traits, guide in optimizing cultivation techniques, and ultimately maximize the quality and quantity of cannabinoid production [6].

Glandular trichomes are structures found on the surfaces of cannabis plants that play a pivotal role in the biosynthesis and accumulation of the bioactive compounds responsible for the plant’s medicinal and recreational properties [13]. The intricate morphology and characteristics of cannabis trichomes have significant implications for the production and quality of cannabinoids, terpenes, and other secondary metabolites [14]. Trichome density, size, and structure directly relate to the potency, flavor, aroma, and therapeutic effects of cannabis products [15]. Furthermore, trichomes are thought to serve as a defense mechanism against environmental stressors and herbivory, acting as both physical barriers and through the production of chemical compounds that deter pests [16]. Understanding the variations in trichome characteristics throughout the cannabis life cycle can provide valuable insights into optimizing cultivation practices, strain selection, and the production of cannabis-based products with consistent and desirable attributes [17]. Therefore, a deeper understanding of cannabis trichome characteristics is crucial for interpreting the plant’s chemical ecology, improving cultivation techniques, and meeting the demands of the growing cannabis industry.

While there is a wealth of research exploring the morphological traits of cannabis, most studies focus on specific growth stages. Thus, a comprehensive investigation of the complete life cycle of specific drug-type cultivars is warranted. Such studies will shed light on the morphological variations that occur from seed germination to flower harvesting.

This study aimed to characterize various morphological traits, including leaf morphology, plant height, and trichome features, throughout the life cycle of a popular drug-type cannabis cultivar, White Widow. By focusing on a specific cultivar, detailed insights into the morphological dynamics and their potential implications for cannabinoid production and plant quality can be achieved. White Widow, a widely popular cannabis cultivar with breeders and connoisseurs, is known to relay potent psychoactive effects. As such, it provides an excellent model to investigate the morphological changes occurring throughout development that are associated with drug-type cannabis [18]. The findings from this study will contribute to the depth of knowledge surrounding the morphological plasticity of cannabis, providing practical and fundamental insight for cultivators and researchers in the cannabis industry. The information presented can be used to inform cultivation practices aimed at improving desirable morphological traits and cannabinoid profiles.

## 2. Results

### 2.1. Leaf Morphology

**Figure 1:**
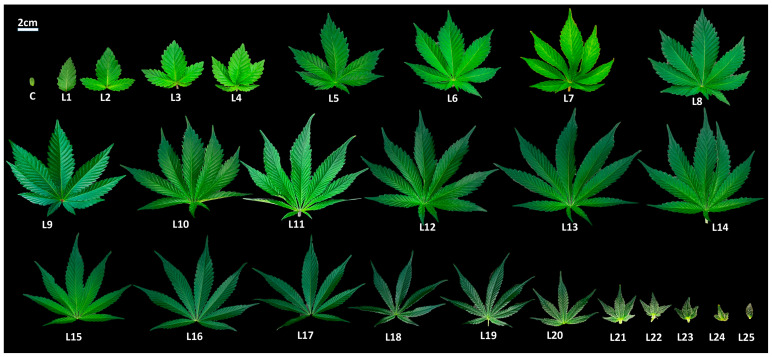
Leaf morphogenesis in cannabis from cotyledon (c) to 25th true leaf (L25) in the main stem. From “c” to “L12,” the plants were maintained under a long-day photoperiod. Starting from L13 and continuing thereafter, the plants were subjected to short-day photoperiod conditions.

This investigation into the morphological characteristics of cannabis leaves during their development from seed germination to maturity revealed a distinct and organized pattern of leaf morphogenesis (Figure 1 and Table S1).

After the expansion of cotyledons, the first true leaves (L1) were each one single serrated leaflet, which served as an initial stage of leaf development. Subsequently, the second true leaves (L2) emerged, featuring three serrated leaflets each, marking a notable increase in leaf complexity. Nodes 3 and 4 produced leaves with five serrated leaflets (L3 and L4), further highlighting the progressive trend in leaflet formation. At nodes 5 and 6, the number of serrated leaflets continued to increase, resulting in leaves with seven (L5) and nine (L6) serrated leaflets, respectively. This developmental sequence depicted in Figure 1 clearly demonstrates the stepwise progression of leaflet complexity during the early stages of cannabis growth.

From node 6 to node 13, the leaves consistently produced nine serrated leaflets each (L6 to L13). This stable phase of leaflet development indicated a period of consistent growth and leaf patterning. It is noteworthy that the photoperiod was shifted from a long-day (16/8) to a short-day (12/12) after L12. However, a shift occurred at node 14, where leaves with eight serrated leaflets (L14) developed. This unique deviation from the previous pattern suggests a transitional phase in leaflet formation. At node 15, leaves reverted to seven serrated leaflets (L15), indicating a return to the standard odd number of leaflets. Nodes 15 to 19, consistently produce leaves with seven leaflets (L15–L19), suggesting a period of stasis in cannabis leaf morphology (Figure 1). The later nodes, from 20 to 24, exhibited a progressive decrease in leaflet complexity, with the number of serrated leaflets decreasing to five (L20–L22) and then further decreasing to three leaflets (L23 and L24). Finally, leaves of one single serrated leaflet (L25) were produced at node 25 (Figure 1).

**Figure 2:**
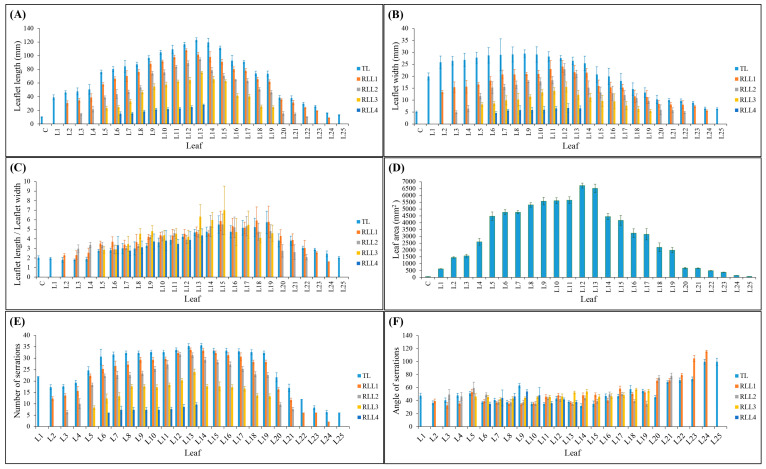
Leaf morphology-related traits including (**A**) leaflet length, (**B**) leaflet width, (**C**) ratio of leaflet length to leaflet width, (**D**) leaf area, (**E**) number of serrations, and (**F**) angle of serrations. Error bars show standard deviation. Error bars show standard deviation. TL: terminal leaflet; RLL1: first right lateral leaflet; RLL2: second right lateral leaflet; RLL3: third right lateral leaflet; RLL4: fourth right lateral leaflet. From “c” to “L12,” the plants were maintained under a long-day photoperiod. Starting from L13 and continuing thereafter, the plants were subjected to short-day photoperiod conditions.

In this study, the morphological changes in cannabis leaflets along the developmental sequence from Leaf 1 (L1) to Leaf 25 (L25) were investigated. The results revealed intriguing patterns of variation in both terminal and lateral leaflet characteristics (Figure 2). As with the leaflet number, the length of the terminal leaflet (Figure 2A) exhibited a progressive increase from L1 (38.96 ± 3.58 mm) to L13 (123.28 ± 3.04 mm), followed by a subsequent decrease from L13 to L25 (13.02 ± 0.69 mm). Furthermore, the length of the first right lateral leaflet exhibited an increase from 30.68 ± 3.2 mm in L2 to 108.05 ± 2.15 mm in L12. Subsequently, there was a gradual reduction observed from L12 to L24, reaching 8.67 ± 0.33 mm (Figure 2A). Similarly, the length of the second right lateral leaflet grew from 14.41 ± 1.36 mm in L3 to 94.73 ± 2.00 mm in L13, followed by a gradual decline from L13 to L22, measuring 10.0 ± 0.57 mm (Figure 2A). The length of the third right lateral leaflet increased from 23.24 ± 2.84 mm in L5 to 75.48 ± 1.7 mm in L13, and then gradually decreased from L13 to L19, measuring 24.5 ± 2.05 mm (Figure 2A). The length of the fourth right lateral leaflet increased from 14.96 ± 2.7 mm in L6 to 27.95 ± 1.26 mm in L13 (Figure 2A). Analogous patterns were likewise noted in the length of all the left lateral leaflets (Table S1).

The width of the terminal leaflet (Figure 2B) exhibited a progressive increase from L1 (19.92 ± 1.48 mm) to L9 (29.49 ± 1.54 mm), followed by a subsequent decrease from L9 to L25 (6.4 ± 0.39 mm). Additionally, the width of the first right lateral leaflet increased from 13.38 ± 0.65 mm in L2 to 24.06 ± 2.5 mm in L12, and then gradually decreased to 5.53 ± 0.36 mm in L24 (Figure 2B). The width of the second right lateral leaflet increased from 4.9 ± 0.62 mm in L3 to 22.92 ± 2.41 mm in L12, and then gradually decreased to 4.84 ± 0.48 mm in L22 (Figure 2B). The width of the third right lateral leaflet increased from 8.2 ± 0.71 mm in L5 to 15.5 ± 2.12 mm in L12, and then, decreased to 5.44 ± 0.68 mm in L19 (Figure 2B). In addition, the width of the fourth right lateral leaflet increased from 4.57 ± 0.57 mm in L6 to 6.7 ± 1.81 mm in L12, followed by a subsequent decrease to 6.53 ± 1.04 mm in L13 (Figure 2B). Similar trends were also observed in the width of all the left lateral leaflets (Table S1).

The ratio of length to width of the terminal leaflet slowly decreased from 1.96 ± 0.12 in L1 to 1.8 ± 0.1 in L3, followed by a gradual increase to 5.5 ± 0.81 in L15 (Figure 2C). After that, the ratio decreased to 4.74 ± 0.64 in L16 which coincides with inflorescence development in the apical regions of the main stem (Figure 2C). Subsequently, the ratio increased to 5.74 ± 1.17 in L19, followed by a gradual decrease in further leaves (2.04 ± 0.13 in L25) (Figure 2C). The ratio of length to width of the first right lateral leaflet increased from 2.29 ± 0.16 in L2 to 3.7 ± 0.52 in L6, followed by a decrease to 3.4 ± 0.46 in L7 (Figure 2C). Subsequently, this ratio gradually increased to 5.88 ± 1.02 in L15, followed by decreasing to 5.16 ± 0.6 in L17, and then increasing to 5.92 ± 1.42 in L18 (Figure 2C). Finally, this ratio gradually decreased to 1.57 ± 0.06 in L24 (Figure 2C). The ratio of length to width of the second right lateral leaflet increased from 2.98 ± 0.39 in L3 to 3.37 ± 0.27 in L4, followed by decreasing to 2.9 ± 0.45 in L6 and increasing again to 4.6 ± 0.33 in L11 (Figure 2C). After a slight decrease (3.93 ± 0.44) in L12, the ratio increased to 5.33 ± 1.02 in L17, followed by decreasing to 2.09 ± 0.29 in L22 (Figure 2C). The ratio of length to width of the third right lateral leaflet exhibited an increase from 2.84 ± 0.34 in L5 to 4.81 ± 0.58 in L9 (Figure 2C). A fluctuation trend was observed across the subsequent leaves (L10 to L19), with their ratio showing a parallel pattern of increase and decrease (Figure 2C). The ratio of length to width of the fourth right lateral leaflet decreased from 3.34 ± 0.89 in L6 to 2.77 ± 0.48 in L7, followed by increasing to 3.81 ± 1.04 in L10 (Figure 2C). After a decrease in L11 (3.48 ± 0.44), the ratio increased to 4.36 ± 0.69 in L13 (Figure 2C). Analogous patterns were likewise noted in the ratio of length to width of all the left lateral leaflets (Table S1). Furthermore, the leaf area increased from 619.98 ± 9.05 in L1 to 6726.69 ± 182.42 in L12, followed by decreasing to 69.96 ± 1.31 in L25 (Figure 2D). This suggests that there exists a critical point in leaf development where growth switches from a positive to a negative trend. This change in the trend coincided with a shift in the phyllotaxy and photoperiod to short-day.

This study also examined the leaf marginal pattern including the number and angle of serration. The number of serrations in the terminal leaflet decreased from 22 ± 0 in L1 to 17.33 ± 1.03 in L2, followed by an increase to 35.67 ± 0.82 in L14. Subsequently, the number of serrations decreased to 6 ± 0 in L25 (Figure 2E). The number of serrations in the first right lateral leaflet increased from 12.33 ± 0.82 in L2 to 33.67 ± 0.82 in L13, followed by decreasing to 2 ± 0.0 in L24 (Figure 2E). The number of serrations in the second right lateral leaflet increased from 6.33 ± 0.82 in L3 to 31.67 ± 0.82 in L12, followed by a decrease to 7.67 ± 0.82 by L21 (Figure 2E). The number of serrations in the third right lateral leaflet increased from 4.17 ± 0.82 in L5 to 24 ± 1.26 in L13, followed by decreasing to 13.33 ± 1.03 in L19 (Figure 2E). The number of serrations in the fourth right lateral leaflet increased from 6 ± 0.0 in L6 to 9.67 ± 0.82 in L13 (Figure 2E). Similar trends were also observed in the number of serrations of all the left lateral leaflets (Table S1).

Although the angle of serration in the terminal leaflet from L1 to L20 had a fluctuating trend, it significantly increased in L21–L25 (Figure 2F). The serration angle within the first right lateral leaflet, spanning from L2 to L19, displayed a fluctuating pattern; however, a substantial increase was observed in the L20–L24 region. Notably, leaflets L23 and L24 exhibited obtuse angles (Figure 2F). The angle of serration in the second right lateral leaflet from L3 to L19 had a fluctuating trend, however, it significantly increased in L20 and L21 (Figure 2F). The angle of serration in the third and fourth right lateral leaflets showed a fluctuating trend (Figure 2F). Analogous trends were also detected in the serration angles of all the left lateral leaflets (Table S1).

**Figure 3:**
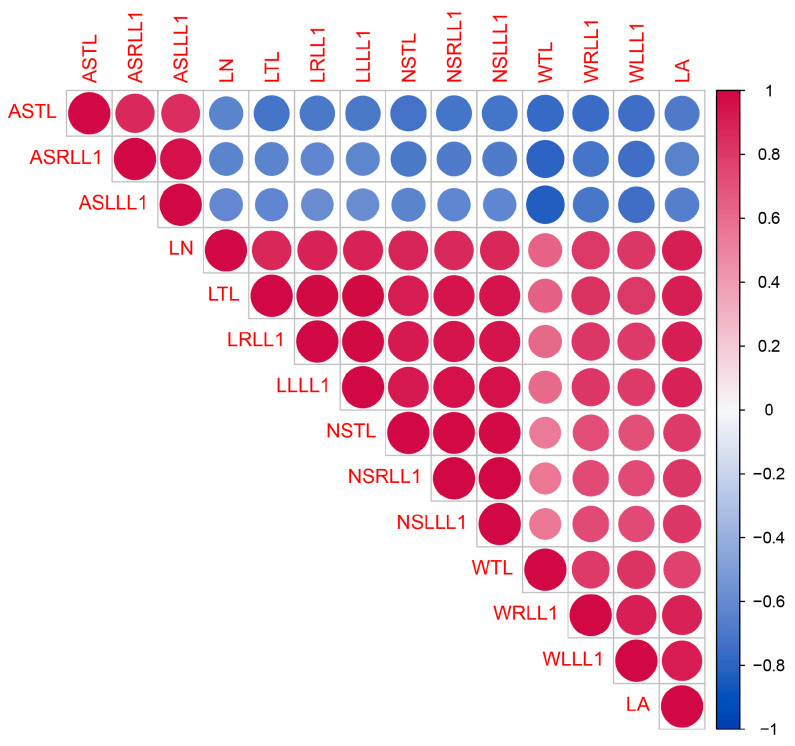
Correlation among morphological traits of cannabis leaf. LA: leaf area; LN: number of leaflets; LTL: length of terminal leaflet; WTL: width of terminal leaflet; LRLL: length of right lateral leaflet; LLLL: length of left lateral leaflet; WRLL: width of right lateral leaflet; WLLL: width of left lateral leaflet; ASTL: angle of serration in terminal leaflet; NSTL: number of serrations terminal leaflet; ASRLL: angle of serration in right lateral leaflet; ASLLL: angle of serration in left lateral leaflet; NSRLL: number of serrations in right lateral leaflet; NSLLL: number of serrations in left lateral leaflet.

A correlation analysis revealed significant positive correlations among all leaf traits, except for the angle of serration (Figure 3). Notably, the angle of serration in both terminal and lateral leaflets (i.e., ASTL, ASRLL1, and ASLLL1) exhibited significant negative correlations with all other traits (Figure 3). However, ASTL, ASRLL1, and ASLLL1 displayed significant positive correlations among themselves (Figure 3).

### 2.2. Plant Architecture

**Figure 4:**
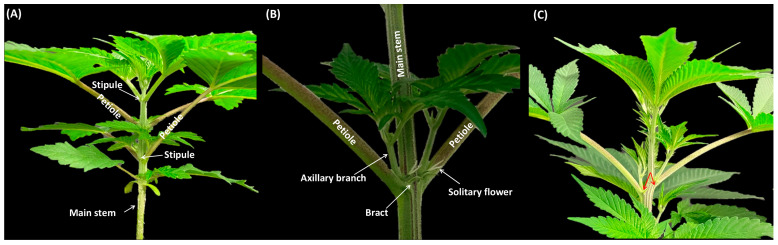
Phase-transition-related morphological changes. (**A**) Cannabis plants with stipules and opposite phyllotaxy, showing the vegetative phase; (**B**) Cannabis plants with bracts and solitary flowers, signifying the transition of the plants into the reproductive phase; (**C**) Cannabis plants with alternate phyllotaxy (red arrows show the alternate arrangement of leaves).

**Figure 5:**
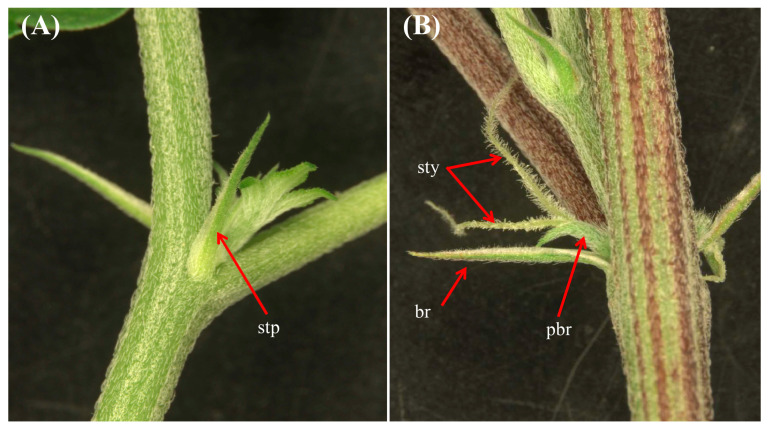
Morphological characteristics of the cannabis plants during the vegetative phase and the reproductive phase. (**A**) Two stipules (small, leaf-like structures found at the base of a petiole), demonstrating the juvenile phase. (**B**) Bracts (modified leaves found just below a flower) and solitary flowers (i.e., perigonal bracts and style), demonstrating the reproductive phase. stp: stipule; sty: style; br: bract; pbr: perigonal bract.

During the growth under the long-day photoperiod, nodes 2–4 exhibited the presence of axillary buds, accompanied by a pair of leaves at each node showing the plants were in the juvenile phase (Figure 4A). Additionally, two stipules were observed flanking the base of the leaf petiole in node 5 and node 6, demonstrating the transition of the plants from the juvenile phase into the mature phase (Figure 4A and Figure 5A).

**Figure 6:**
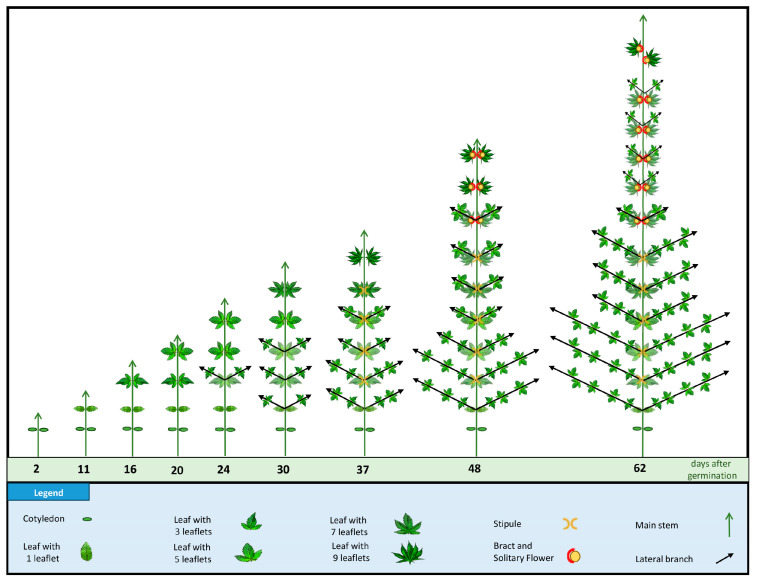
Plant growth and development under long-day photoperiod.

Relating to nodes 7–12, the discernible elements included foliar leaves (sustained by petioles), bracts, and solitary flowers, collectively signifying the transition of the plants into the reproductive phase (Figure 4B and Figure 5B). Nodes 1 through 11 showed opposite leaf arrangements (Figure 4A,B), followed by a shift in phyllotaxy characterized by the transition to alternate leaf arrangement starting with node 12, which continued in subsequent nodes (Figure 4C). Node 1 of the axillary shoots produced a pair of leaves with three serrated leaflets, while 2 onward in the axillary shoots produced a pair of leaves with five serrated leaflets (Figure 6), all of which were in the opposite phyllotaxy (Figure 6).

**Figure 7:**
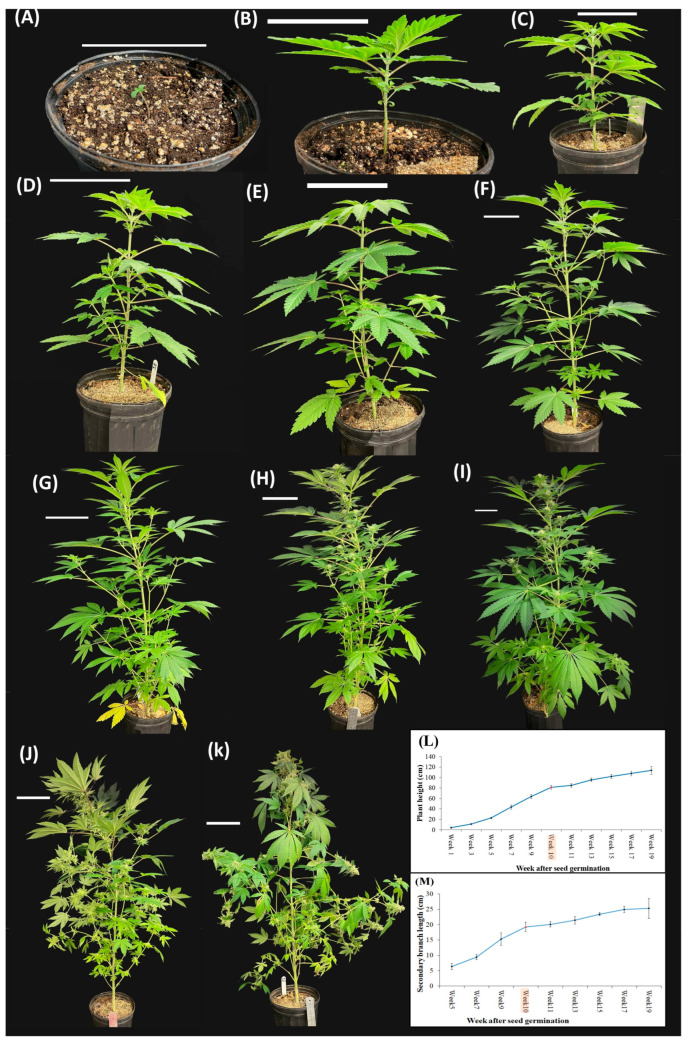
Plant growth and development during the complete life cycle including: (**A**) 1 week, (**B**) 3 weeks, (**C**) 5 weeks, (**D**) 7 weeks, (**E**) 9 weeks, (**F**) 10 weeks, (**G**) 11 weeks, (**H**) 13 weeks, (**I**) 15 weeks, (**J**) 17 weeks, and (**K**) 19 weeks after seed germination. Scale bar = 10 cm; (**L**) Plant height during the complete life cycle of cannabis plants; (**M**) The length of the longest secondary branch of cannabis plants during the complete life cycle.

There was substantial growth related to plant height associated with the long-day photoperiod (18/6). During the long-day photoperiod, the plant height increased from 4.05 ± 0.62 cm in seedlings with cotyledons to 81.23 ± 3.51 cm in plants with the twelve true leaves (Figure 7). In addition, the longest secondary branches in the lower part of the plant extended to a maximum length of 19.28 ± 1.52 cm (Figure 7).

**Figure 8:**
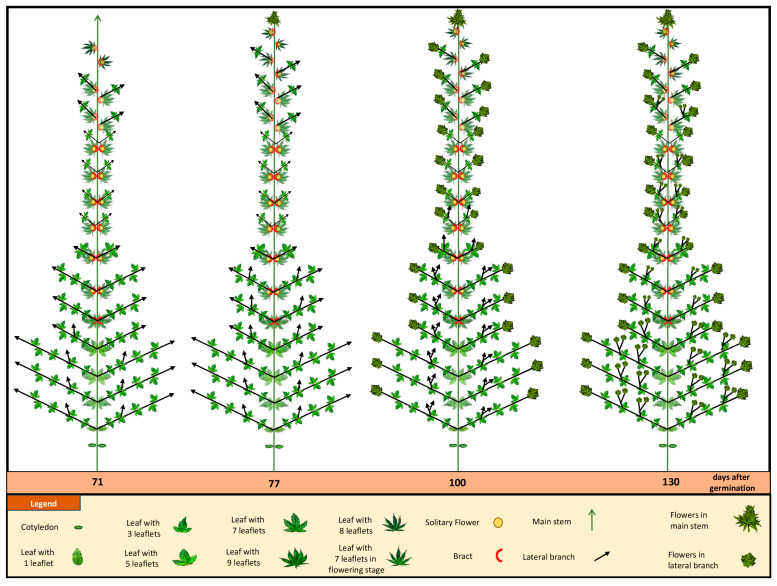
Schematic representation of plant growth and development under short-day photoperiod.

Once the phyllotaxy shifted from opposite to alternative, the photoperiod was changed to short-day conditions (12/12). To prevent the potential misidentification of solitary flower stigmata within the apical zone as inflorescences, the commencement of inflorescence development was delineated as the point where a minimum of three pairs of stigmata became discernible atop the apical shoot. After 10 days of the short photoperiod, inflorescence was observed atop the apical shoot at node 15. At the full-flowering stage (after 15 days of the short-day photoperiod), the main inflorescences became evident at the apical extremity of both the main stem and secondary- and tertiary-order branches (Figure 7 and Figure 8). Moreover, all leaves of the main and axillary shoots were in an alternate phyllotaxy. The plant height in the short-day photoperiod increased from 81.23 ± 3.51 cm to 113.62 ± 7.58 cm (Figure 7). In addition, the longest secondary branches in the lower part of the plant extended to a maximum length of 25.31 ± 3.24 cm (Figure 7). It is notable that the augmentation of plant height and secondary branch length in the short-day photoperiod exhibited reduced growth compared to the long-day photoperiod (Figure 7).

### 2.3. Floral Morphology

**Figure 9:**
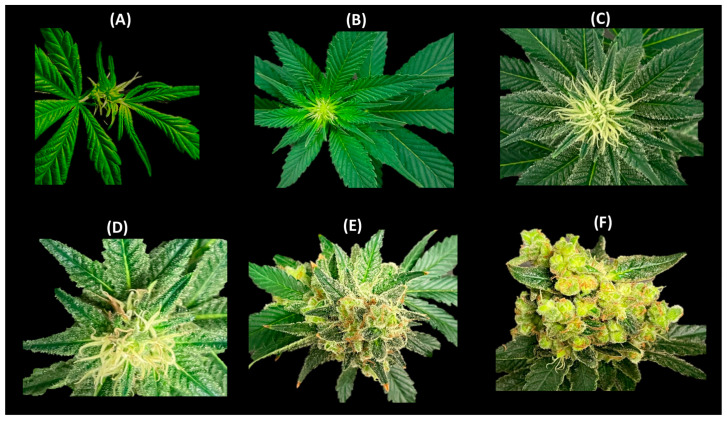
Flowering progress in cannabis. Main flower after (**A**) 3 days, (**B**) 1 week, (**C**) 2 weeks, (**D**) 4 weeks, (**E**) 6 weeks, and (**F**) 8 weeks.

Upon transition to the short-day photoperiod, compacted inflorescences emerged at the apical regions of the main stem (after 10 days) and secondary- and tertiary-order branches (after 15 days). Cannabis inflorescences presented as complexly branched compound racemes. This distinctive structure is emblematic of monopodial growth, featuring an enduring apical meristem and indeterminate axillary inflorescences of higher orders. Flower development at the apical regions of the main stem is shown in Figure 9. During flower development, prior to stigma elongation, a profusion of glandular trichomes emerged on the perigonal bract encasing the ovary. As the inflorescences underwent maturation/senescence, the color of the stigmas transitioned from a whitish-yellow hue to a reddish-brown shade (Figure 9).

**Figure 10:**
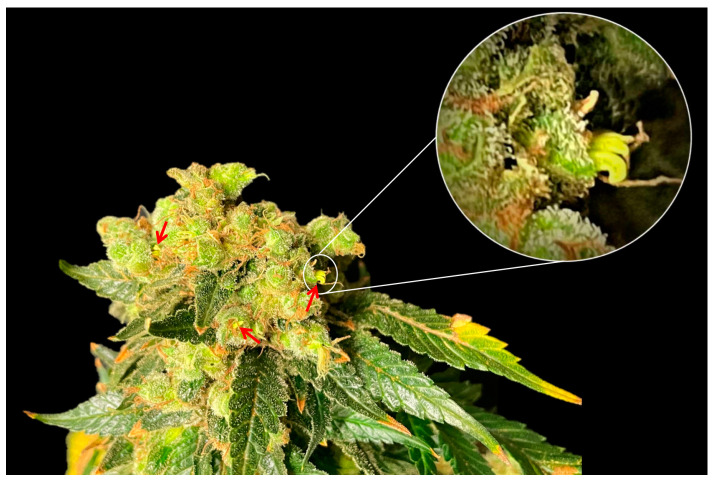
Hermaphroditic cannabis inflorescence. Red arrows show male flowers.

Female cannabis inflorescences manifested in pairs of florets within the leaf axils, corresponding to the initial, smaller branchlets of the secondary axillary branch that emerges between them. Indeed, every inflorescence equated to compacted higher-order branchlets, each of which preserved an identical phytomer structure akin to the larger phytomers produced during long-day photoperiods. These condensed branchlets contained single leaflet leaves, an axillary shoot, one or two solitary flowers, and bracts (Figure 9). It is notable that, under the experimental conditions of the current study, male florets were observed in some of the plants after 140 days (Figure 10), which was well after the ideal harvesting date.

### 2.4. Trichome Development

**Figure 11:**
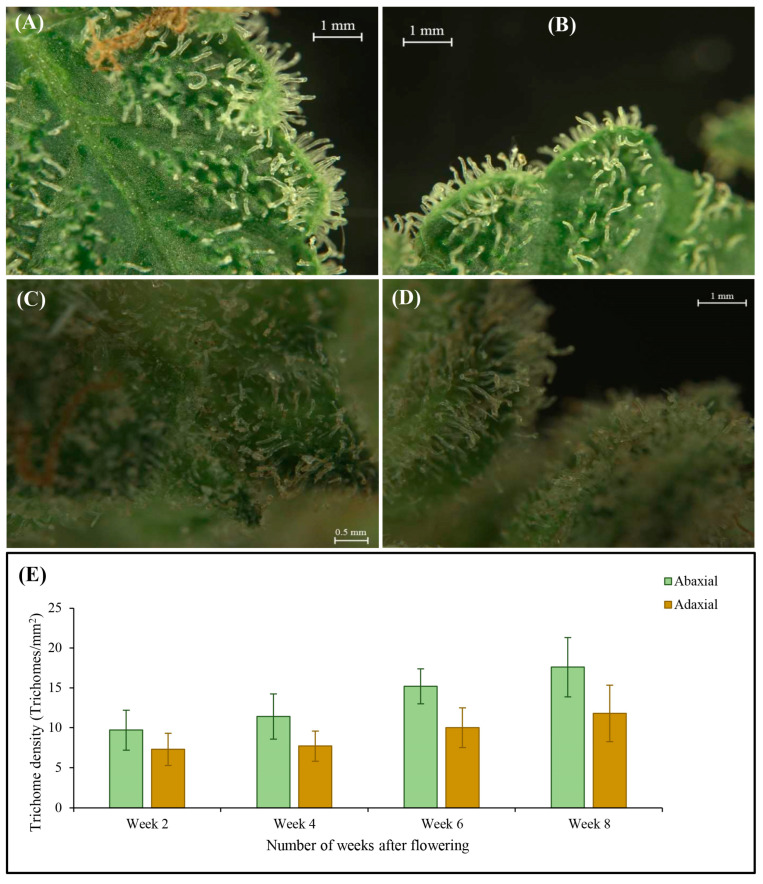
Trichome distribution in the upper portion of the bracts after (**A**) 2 weeks of flowering or 86 days of seed germination, (**B**) 4 weeks of flowering or 100 days of seed germination, (**C**) 6 weeks of flowering or 114 days of seed germination, and (**D**) 8 weeks of flowering or 128 days of seed germination; (**E**) Trichome density during flower development.

Glandular trichome density began to increase shortly after the photoperiod was switched to short-day conditions. The density of glandular trichomes on both the abaxial and adaxial sides of the bract demonstrated an increase from week 2 to week 8 (Figure 11A–D) following the shift to short-day photoperiod. However, a higher density of trichomes was discernible on the abaxial surface of the bracts (Figure 11E).

**Figure 12:**
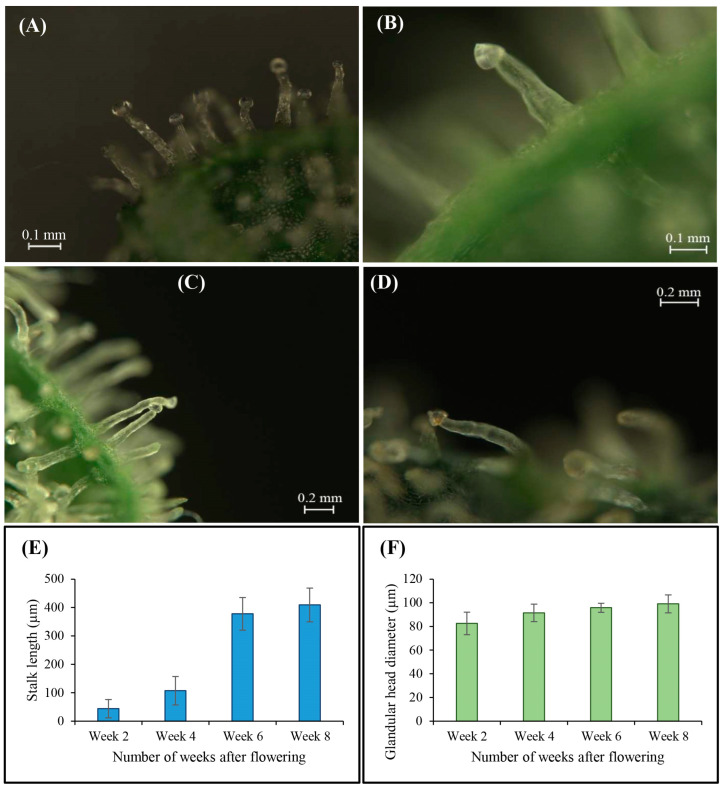
Glandular trichome after (**A**) 2 weeks of flowering or 86 days of seed germination, (**B**) 4 weeks of flowering or 100 days of seed germination, (**C**) 6 weeks of flowering or 114 days of seed germination, and (**D**) 8 weeks of flowering or 128 days of seed germination; (**E**) Trichome stalk length during flower development; (**F**) Glandular head diameter during flower development.

In the temporal evolution of trichome development spanning from week 2 to week 8 of the short-day photoperiod (Figure 12A–D), there was a concurrent augmentation observed in both trichome stalk length (Figure 12E) and glandular head diameter (Figure 12F). This developmental progression correlates with the transition of resin color within maturing glandular heads on trichomes, transitioning from its initial transparent or translucent state (Figure 12A) to a later opaque (milky white) appearance (Figure 12B,C), culminating in a final amber (brown) shade (Figure 12D).

**Figure 13:**
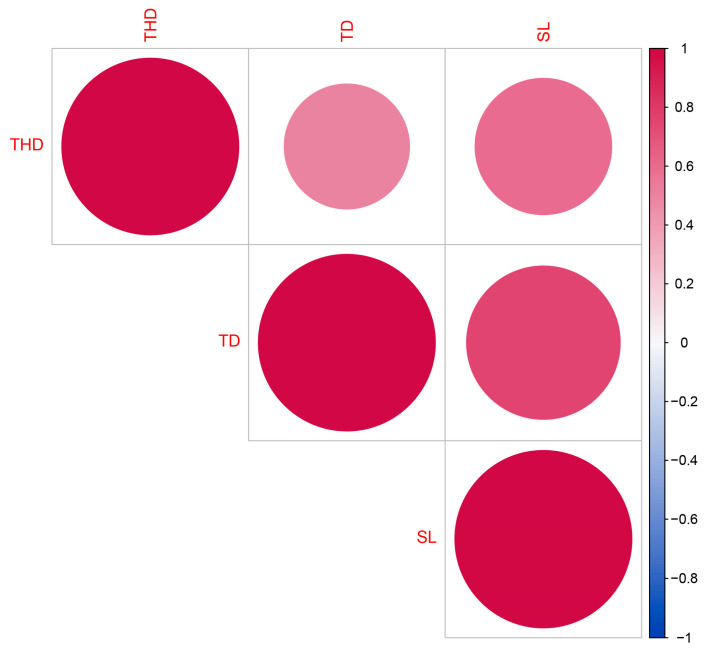
Correlation among morphological traits of cannabis trichome. TD: trichome density; THD: trichome head diameter; SL: stalk length.

A correlation analysis was completed based on trichome traits (Figure 13). The measurable traits include trichome density, trichome stalk length, and glandular head diameter. The analysis revealed significant positive correlations among all trichome traits listed.

## 3. Discussion

Cannabis is known to exhibit considerable variation in growth habits, leaf morphology, flowering time, cannabinoid content, and secondary metabolite synthesis throughout the plant life cycle [8,19]. Understanding the mechanisms driving such morphological changes in cannabis is essential for optimizing cultivation practices and harnessing the plant’s bioactive compounds for medicinal and industrial purposes [6,11,20,21]. While several studies [6,11,17,21] have compared cannabis morphology in various contexts, this study is fundamentally different, delving into the ontological development of cannabis to provide a comprehensive examination of the morphological characteristics emerging throughout its life cycle. This holistic approach not only enhances our understanding of this plant but also holds several implications for horticultural practices. This type of study unravels the intricate biological processes governing growth and development to shed light on the emergent transitions and adaptations undergone throughout the cannabis life cycle [22]. Insight into the plant’s morphological changes can serve as a valuable roadmap for horticulturists seeking to optimize cultivation practices. For instance, understanding the stages of juvenility in cannabis is crucial for improving rooting success, facilitating micropropagation establishment, and enhancing overall plant vigor. Additionally, understanding the process of transition to maturity and floral development can guide commercial practices for the production of floral biomass.

Moreover, the findings of the current study hold immense potential to advance the broader field of cannabis research. The morphological clues presented provide the foundation for further investigations related to gene regulation and transcriptome profiles at different developmental stages of the cannabis life cycle. This fundamental knowledge is indispensable for conducting more sophisticated studies on the genetic underpinnings of cannabis biology, enabling researchers to explore the plant’s relevance to various applications, from medicinal to industrial, with greater precision and efficacy.

Cannabis undeniably showcases some of the most recognizable leaves in the botanical realm [7]. Characterized by palmately compound structures encompassing a diverse range of leaflet numbers, these leaves have ingrained themselves as symbols within popular culture [7]. The extensive range of leaf morphology has been previously documented by Quimby et al. [23], and subsequent work by Anderson [24] provided the pioneering quantification of central leaflet dimensions, including length, width, and proportional ratios.

The intriguing developmental pattern observed in the number of leaflets within cannabis leaves represents one of the more interesting morphological properties presented. The transition from single leaflet leaves to leaves with nine leaflets during the mature stage, then reversion back to leaves of one leaflet during the flowering stage, prompts a comprehensive investigation into the underlying factors governing this phenomenon. The shift in leaflet count reflects a dynamic response to the plant’s developmental requirements and environmental cues [25,26].

During the early stages of growth, the gradual increase in the leaflet number, length, and width of terminal and lateral leaflets, as well as leaf area, can be attributed to the plant’s strategy to maximize photosynthetic efficiency and resource capture [9]. The progression to leaves with nine serrated leaflets (L6 to L13) indicates a stable phase of optimized photosynthesis, suggesting that the plant has reached an equilibrium between energy production and consumption [9]. This developmental phase could also be influenced by hormonal changes that dictate leaf patterning and growth [25,26].

The subsequent reduction in leaflet number, length, width of terminal and lateral leaflets, and leaf area as the cannabis plant transitions to its flowering stage may be attributed to the reallocation of resources towards reproductive structures. The energy-intensive process of flowering necessitates a redirection of nutrients and resources towards flower and seed production, potentially leading to a reduction in leaf complexity [9]. The shift from leaves with nine serrated leaflets (L6 to L13) to leaves with decreasing leaflet numbers (L14 to L25) and overall leaf area suggests a trade-off between vegetative growth and reproductive efforts [17]. The reversion to leaves with a single serrated leaflet (L25) at the flowering stage could be explained by the plant’s prioritization of reproductive success over vegetative expansion [9]. Alterations in morphological traits of cannabis leaves throughout various developmental stages exhibit congruence with observations in diverse plant species [8]. This consistency underscores fundamental patterns in leaf development that extend beyond taxonomic boundaries. For instance, the increase in the number of leaflets observed in cannabis during vegetative growth is analogous to species such as hairy bittercress and tomato, revealing a shared mechanism governing leaflet proliferation during this phase [27,28,29]. Furthermore, the changes in leaf size and shape observed during cannabis’ transition from the vegetative to reproductive stages mirror the phenomena documented in other plant species, emphasizing the broader applicability of these findings and suggesting a conserved regulatory framework governing leaf morphogenesis in plants [17,25,26]. This cross-species consistency in leaf morphological traits enhances the general understanding of cannabis development while contributing to the broader knowledge of plant biology.

Under long-day photoperiod conditions, the developmental progression of cannabis plants is distinctly outlined. Nodes 2–4 displayed the presence of axillary buds and leaves, indicating the juvenile phase. Nodes 5 and 6 exhibited the emergence of stipules flanking the base of the leaf petiole, signifying the transition from juvenility to maturity. The shift to the reproductive phase was marked by nodes 7–12, and characterized by distinct elements such as foliar leaves, bracts, and solitary flowers (colloquially referred to as pre-flowers). In line with the results of the current study, previous studies showed that, under long-day photoperiod conditions, cannabis plants may undergo differentiation to show initial solitary flowers within the fourth to seventh internodal regions [17,22,30]. The shift in phyllotaxy from an opposite to an alternate arrangement further signifies a developmental shift in the plant [30]. The change in leaflet count and phyllotaxy in the axillary shoots emphasizes the dynamic nature of leaf development during different growth stages [10]. Furthermore, the current investigation presents information that can be used for cannabis rejuvenation through tissue culture. It is well-documented that tissue culture techniques can effectively rejuvenate aging plants, prompting them to revert to a juvenile state, often accompanied by the re-establishment of characteristics such as opposite leaf arrangement [2,20]. This capability has profound implications for plant propagation, the maintenance of genetic integrity, and the potential enhancement of overall crop yields [20,31].

The emergence of solitary flowers during long-day conditions has complicated the characterization of the phase transition from the vegetative to reproductive stages in cannabis. Despite the designation of a long-day photoperiod as ‘non-inductive’ for cannabis, the presence of solitary flowers under a long-day photoperiod shows a phase transition in cannabis plants [17]. The initiation of these solitary flowers is believed to be influenced by the plant’s age and regulated by intrinsic cues rather than being governed by the photoperiod [22]. Since solitary flowers can manifest under both extended and shortened photoperiods, it has been suggested that cannabis displays a day-neutral response concerning this facet of flower induction [22]. However, the question of whether the initiation of solitary flowers marks the culmination of the vegetative phase remains unresolved. Vegetative development may persist at the shoot apical meristem (SAM) during the interval between the appearance of solitary flowers and the ultimate induction of inflorescence development [30].

In the current study, the transition to a short-day photoperiod initiated a distinct set of morphological changes. The precise recognition of inflorescence onset, marked by the appearance of multiple pairs of stigmas, allowed the accurate delineation of the inflorescence initiation phase. After 10 days of short-day photoperiod exposure, inflorescences became evident at node 15. At full flowering (15 days of short-day photoperiod), the main inflorescences were observed at the apical extremities of the primary and secondary branches. The onset of inflorescence development is characterized by alterations in the structural configuration of the shoot apex [17]. This transformation results in the development of a complex raceme featuring densely branched appendages and recurrent phytomer structures. These phytomer formations encompass an internode, a foliar leaf (sustained by a petiole), bracts, and solitary flowers (comprising the stigma, style, and perigonal bract) [17].

The intricacy of morphophysiological attributes linked to flowering patterns in cannabis has resulted in the use of variable terms when documenting these characteristics [5,17,32]. Previous investigations have postulated the occurrence of four primary events during florogenesis: (1) the initiation of solitary flowers, (2) the emergence of axillary branches and the shift towards more complex branching structures, (3) the initiation of inflorescences, characterized by the formation of flower clusters at both the shoot apex and axillary branches, and (4) terminal flowering, signifying the transformation of the apical meristem into a terminal flower [22,30]. Alterations in the shoot apex architecture and the onset of inflorescence flowering have demonstrated inducibility by short-day photoperiods, with evidence suggesting that these traits are regulated independently from the formation of solitary flowers [17].

Upon exposure to the short-day photoperiods, cannabis plants transitioned to the reproductive phase, characterized by the emergence of compacted inflorescences in the apical regions of both the main stem, secondary-, and tertiary-order branches. The complex branched raceme structure of the cannabis inflorescence is a hallmark of monopodial growth, featuring a persistent apical meristem and indeterminate axillary inflorescences of higher orders [17]. This branching pattern reflects the plant’s strategy to maximize floral development potential through repeated branching and reiteration, enabling the production of numerous reproductive structures [17]. The emergence of glandular trichomes on the perigonal bracts encasing the ovary during flower development signifies the initiation of resin production, a characteristic feature of cannabis flowers [15]. The transformation of the stigma color from whitish-yellow to reddish-brown as the inflorescences mature/senesce underscores the physiological changes that occur during flower development. This transition may correspond to the maturation of the flower [14,15]. The presence of paired female inflorescences within the leaf axils, associated with smaller branchlets of secondary axillary branches, demonstrates the branching pattern of cannabis inflorescences and the consistent phytomer structure of the higher-order branchlets [17].

Sexual dimorphism (i.e., the distinct physical and physiological differences between males and females of a species) is a fascinating and intricate phenomenon in cannabis [33]. Cannabis exhibits a karyotype composed of a single pair of heteromorphic sex chromosomes and nine pairs of homomorphic autosomal chromosomes [34]. Cannabis plants, especially genotypes used for medicinal/recreational purposes, generally display dioecious attributes, characterized by well-defined male and female individuals [34]. Nonetheless, the inherent flexibility in sexual expression can give rise to the emergence of hermaphroditic plants, recognized as monoecious phenotypes [33]. Female cannabis plants have pistillate flowers densely clustered and interspersed with leafy bracts [33]. Some dioecious plants are known to become hermaphroditic and produce male flowers [33]. An interesting observation in the current study was the appearance of male flowers in various plants after a prolonged period. These flowers only appeared long after the plants would have normally been harvested in a commercial setting. Specifically, male flowers were observed at day 76 of the short-day photoperiod when this cultivar is general considered mature at day 56 of the short-day photoperiod. The emergence of male flowers in the late stages of senescence in an unpollinated female plant is a logical adaptation by the plant as a last effort to produce seeds. The late-stage transition into hermaphroditism underscores the genetic diversity and potential for both male and female reproductive structures to emerge from within the same plant, implications of which can be used to guide cultivation practices [33,35].

The investigation of trichome density on both the abaxial and adaxial sides of the bract revealed a notable increase from week 2 to week 8. This temporal trend suggests that, as the plants progressed in age, there was consistent generation of glandular trichomes, which are pivotal sites for resin synthesis and cannabinoid production. Remarkably, higher trichome densities emerged on the abaxial surfaces of the bracts. This asymmetry in trichome distribution might be attributed to the varied environmental conditions affecting the two surfaces differently or could be a result of genetic predisposition influencing the trichome placement [15]. Consistent with the results of the current study, Punja et al. [15] documented increased trichome density on the abaxial surface of bracts. Since trichomes represent major sites of secondary metabolite synthesis and sequestration, this temporal increase in trichome density aligns with the general understanding that secondary metabolite levels peak around the time that plants are considered mature.

The observed evolution of trichome development from week 2 to week 8 unveiled a synchronized enhancement in the trichome stalk length and glandular head diameter. This co-occurring increase suggests that, as trichomes matured, they underwent structural changes that led to the elongation of stalks and the expansion of glandular heads. Importantly, this developmental progression is correlated with color changes within the glandular heads. The shift from a transparent (translucent) initial state to an opaque (milky white) appearance, and, eventually, to an amber (brown) shade, indicates the synthesis and accumulation of cannabinoids and other resinous compounds within the glandular trichomes. This alteration in resin color could signify the maturation and chemical transformation of secondary metabolites, including cannabinoids and terpenes, within the trichomes [13,15,16]. These compounds play crucial roles in the biological and therapeutic properties of cannabis [13].

## 4. Materials and Methods

### 4.1. Plant Material and Growth Conditions

This research involved the cultivation of 25 feminized *Cannabis sativa* L. cv. White Widow plants (Dutch Passion, Netherlands), spanning from the initial seed germination stage to the flower harvesting. Seeds were sown in 25 cm diameter pots containing ProMix substrate (sphagnum peat moss [82–88%], perlite [13–17%], ethoxylated alkylphenol, dolomitic and calcitic limestone, pH = 5.5, and EC = 1.4 mmhos/cm) under controlled conditions in a phytotron. The environmental parameters were maintained at a temperature range of 24–24.5 °C and a relative humidity between 55 and 65%. LED lighting, in the form of Grow 10 Light E1-300W fixtures, emitting a broad “white” spectrum light (with 4.2% red in the 650–670 nm range) was used, delivering 440 μmol/m^2^/s at canopy height. The plants were subjected to a long-day photoperiod (16 h of light followed by 8 h of darkness) until they naturally transitioned to alternate leaf phyllotaxy, which occurred after 62 days under these conditions. Subsequently, the photoperiod was adjusted to a short-day cycle (12 h of light followed by 12 h of darkness). To assess the natural developmental patterns of the plants, no pruning or training of any kind was conducted. Likewise, no defoliation was conducted, with the exception of removing dead or unhealthy leaves. No pesticides were applied, but standard biological controls were used. Plants were watered with reverse osmosis water mixed with 200 ppm of Plant-Prod Complete fertilizer (Table 1). The water/fertilizer combination was relayed directly to the rhizosphere of plants via drip irrigation. The drip irrigation system was set to a timer, which allowed water/fertilizer delivery 2 times per day for 2 min each time (4 min per day total), with 2 min watering cycles occurring every 12 h.

### 4.2. Leaf-Related Morphological Traits

**Figure 14:**
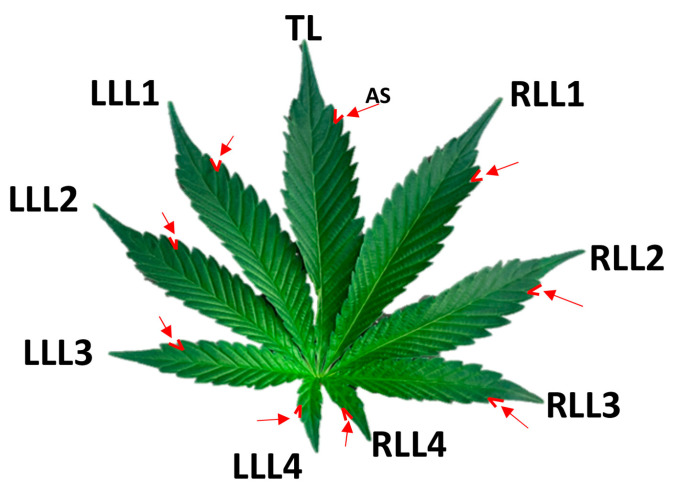
Cannabis-leaf-related morphological traits. Red arrows show angle of serration. AS: Angle of serration; TL: terminal leaflet; RLL1: first right lateral leaflet; RLL2: second right lateral leaflet; RLL3: third right lateral leaflet; RLL4: fourth right lateral leaflet; LLL1: first left lateral leaflet; LLL2: second left lateral leaflet; LLL3: third left lateral leaflet; LLL4: fourth left lateral leaflet.

Throughout the entire plant life cycle, morphological data pertaining to the youngest fully expanded leaf were recorded (Figure 14). These data encompassed various parameters, including leaf arrangement, the number of leaflets, dimensions of the terminal and lateral leaflets (length and width), the number and angle of serrations on the terminal and lateral leaflets, leaf area, and the presence of florets. This data collection was performed using six leaves (six replications) from six randomly chosen plants at each time point to ensure accurate and comprehensive analyses. In this study, image processing techniques were employed to quantify leaf-related morphological traits. The imagery of leaves was acquired using an iPhone Xs equipped with auto-focus capabilities. Previous studies have validated the utility of smartphones for capturing images to investigate plant morphological characteristics [36,37]. Subsequently, the acquired images underwent comprehensive analysis using ImageJ 1.53e software [38].

### 4.3. Plant Architecture Traits

Throughout the entire plant life cycle, biweekly photos were taken of the plants using an iPhone Xs equipped with auto-focus capabilities. This data collection was performed using six randomly chosen plants (six replications) each week. Subsequently, the acquired images underwent comprehensive analysis using ImageJ 1.53e software [38] to measure plant height and axillary stem length.

### 4.4. Flower and Trichome Features

Individual inflorescences (flower buds) were harvested biweekly, specifically within the 2-to-8-week timeframe following the short-day photoperiod, from the apical regions of the main stem. For subsequent analysis, the upper portions of the bracts were carefully excised using a sharp razor blade. A ZEISS Axio Zoom.V16 stereomicroscope was used for measuring trichome density, trichome stalk length, and glandular head diameter.

### 4.5. Data Analysis

Image processing procedures were followed by data analysis. All morphological data were analyzed using AllInOne R-Shiny package (Version 1.9.5) [39]. This included leaf-related morphological data, plant height, axillary stem length, trichome density, trichome stalk length, and glandular head diameter.

## 5. Conclusions

The current study offers a detailed characterization of the entire life cycle of *Cannabis sativa* L. cv. White Widow, encompassing key aspects such as leaf morphology, plant structure, flower development, and trichome features. By studying the whole life cycle, a dynamic progression of the developmental cues and responses of the cannabis plant are revealed. The intricate journey of cannabis leaf development is highlighted by a gradual increase in morphological complexity, from the initiation of L1, one single serrated leaflet, to the peak of nine leaflets characteristic of L6. The leaflet number remained stable from nodes 6–13, which produced leaves with nine leaflets. However, a reduction in leaflet number was observed at node 14, which produced leaves with eight leaflets, and leaves with seven leaflets emerging from node 15. Towards nodes 20–24, the leaf complexity diminished, ultimately resulting in leaves of one single leaflet at node 25. The significant observation of leaf area peaking at L12, and the sharp decline in leaf area thereafter, which also coincides with a change in phyllotaxy, underscores the phase transition of cannabis. Under the long-day photoperiod, the plant’s transition from maturity traits to reproductive traits is marked by the emergence of bracts and solitary flowers. An intriguing shift in leaf arrangement from opposite to alternate was noted at node 12 while still under the long-day photoperiod. The transition to the short-day photoperiod triggered complex raceme-like inflorescence formation at the apical regions, indicative of monopodial growth.

The investigation into flower development revealed abundant glandular trichomes on perigonal bracts, with a dynamic shift in stigma color during maturation. The trichome development exhibited a concurrent growth in stalk length and glandular head diameter, which mirrored the resin’s color shift from transparent to opaque and, ultimately, amber. It is critical that future experiments focus on investigating the genetic and hormonal mechanisms involved in the intricate developmental processes described. In addition to guiding future experiments, the presented findings have significant implications for horticulturists and cultivators seeking to maximize plant health and optimize propagation and cultivation techniques.

The comprehensive developmental roadmap presented in the current study serves as a crucial tool for evaluating and harnessing morphological traits and should be extended to additional cannabis cultivars. By providing a detailed account of the plant’s ontological development, this study enables researchers and cultivators to make informed decisions relating to applicable methods and the timing of implementation. The information provided can inform strategies for plant rejuvenation, propagation, and cultivation, thereby contributing to optimal production outcomes. Moreover, this developmental roadmap is of paramount importance for developing an ontological transcriptomic reference for *Cannabis sativa*. A deeper understanding of the plant’s developmental stages and phase transitions is instrumental in deciphering the underlying genetic mechanisms governing juvenility and other critical traits, such as flowering. The occurrence of the unique solitary flower arrangement, which is separate from inflorescence in cannabis, suggests that the regulatory processes involved in flower induction may differ from other species. Consequently, this study contributes to the foundational knowledge required to unravel the intricacies of cannabis biology, particularly in the context of flowering, which will ultimately facilitate advancements in cultivation techniques.

## Figures and Tables

**Table 1:** The ingredients of Plant-Prod Complete fertilizer.

Ingredient	Concentration	Ingredient	Concentration
Total Nitrogen (N)	17%	Iron (Fe)	0.25%
Nitrate Nitrogen	12.4%	Manganese (Mn)	0.075%
Ammoniacal Nitrogen	4.6%	Zinc (Zn)	0.075%
Available Phosphoric Acid (P_2_O_5_)	5%	Copper (Cu)	0.050%
Soluble Phosphorus (P)	2.1%	Boron (B)	0.020%
Soluble Potash (K_2_O)	17%	Molybdenum (Mo)	0.0015%
Soluble Potassium (K)	14.1%	EDTA	1.48%
Calcium (Ca)	3%	DTPA	0.95%
Magnesium (Mg)	1%	EDDHA	0.10%

## Data Availability

All data generated or analyzed during this study are included in this published article, as Supplementary Information Files.

## Supplementary Materials

The following supporting information can be downloaded at: https://www.mdpi.com/article/10.3390/plants12203646/s1, Table S1: Leaf-related morphological traits of *Cannabis sativa* L. cv. White Widow.
